# 

**Published:** 1857-06

**Authors:** 


### BOOKS, &C., RECEIVED.

We have received from the publishers, Messrs. Blanchard & Lea, of Philadelphia, through Messrs. J. J. Richards & Co., of this city, The Physiological Anatomy and Physiology of Man, by Todd & Bowman, complete in one volume with 298 illustrations,—Ludlow's Medical Examinations—and a new Edition of Churchill on the Diseases of Women, including those of Pregnancy and childbed, revised by the Author, and with notes and additions, by D. Francis Condie, M. D., of Philadelphia, with sundry pamphlets, &c., which will be reviewed in the next number of our Journal, our engagements being of such a character that it is impossible to do them justice at present.

In this connection, we would remark, that books may be sent to us, through Messrs. J. J. Richards & Co., of Atlanta, whose establishment we would recommend to our friends as offering inducements in the way of *supply* and *terms* upon which their books are offered.

### TO CORRESPONDENTS.

☞ All Communications pertaining to the Editorial Department of the Journal, should be addressed to the Editors.

☞ All Communications, having reference to the Subscription, the Advertising or the Financial Department, should be addressed to the Treasurer, J. G. WESTMORELAND, M. D., Dean of the Faculty of the Atlanta Medical College.

